# Mild traumatic brain injury presenting with delayed intracranial hemorrhage in warfarin therapy: a case report

**DOI:** 10.1186/s13256-015-0652-2

**Published:** 2015-08-18

**Authors:** Pearl Chung, Fary Khan

**Affiliations:** The University of Melbourne, Department of Rehabilitation Medicine, Royal Melbourne Hospital, 34-54 Poplar Road, Parkville, Melbourne, VIC 3052 Australia

**Keywords:** Mild traumatic brain injury, mTBI, Mild head injury, Delayed intracranial hemorrhage, Anticoagulation, Warfarin

## Abstract

**Introduction:**

Current literature estimates the risk of delayed intracranial hemorrhage as between 0.6 and 6% after mild head injury for patients on warfarin. Due to resource allocation issues, the need to actually diagnose delayed intracranial haemorrhage has been questioned, especially if it does not require surgery. The purpose of our case report is to consider the functional implications during the six months following a mild traumatic brain injury complicated by delayed intracranial hemorrhage in a patient undergoing warfarin therapy. To the best of our knowledge, the rehabilitative and functional considerations of delayed intracranial haemorrhage in head injury have not been previously described in the literature.

**Case presentation:**

A previously independent 74-year-old Lebanese man living in Australia sustained mild traumatic brain injury following an unwitnessed fall from the height of two meters while on warfarin therapy, with an international normalized ratio of 4.2. He was found to have amnesia of the event and extensive facial bruising. His Glasgow Coma Scale score was 14 to 15 throughout observation. Following a non-diagnostic initial computerised tomography scan, a repeat scan at 24 hours from the injury identified large intracerebral, subdural and subarachnoid hemorrhages. A detailed examination demonstrated visuospatial and cognitive impairments. He required inpatient rehabilitation for three weeks, and outpatient rehabilitation for two months. By six months, he had returned to his pre-injury level of functioning, but was unable to resume driving.

**Conclusions:**

We describe rehabilitation outcomes of delayed intracranial haemorrhage and mild traumatic brain injury, with diminishing disability over six months. In our case report, the complication of the delayed intracranial haemorrhage resulted in significant activity limitations and participation restrictions, which affected the clinical management, including the need for multidisciplinary rehabilitation. The risk of delayed intracranial haemorrhage in mild head injury remains a significant problem requiring further research.

## Introduction

Following a mild head injury, between 0.6 and 6% of people undergoing warfarin therapy may develop delayed intracranial hemorrhage (DICH), despite a non-diagnostic initial computerised tomography (CT) scan of the brain [1–4]. The overall incidence of mild traumatic brain injury (mTBI) in older adults aged 65 and over is estimated to be as high as 537 per 100,000 person-years, of which 509 per 100,000 person-years are due to falls [5]. The definition for mTBI is widely variable in the literature, making the evidence synthesis difficult [6, 7]. For someone who sustains a TBI, as evidenced by an alteration in the brain function caused by an external force [8], a mTBI could be defined as: a Glasgow Coma Scale (GCS) score of 13 or above, any loss of consciousness (LOC) lasting less than 30 minutes and any post-traumatic amnesia (PTA) lasting less than 24 hours [6, 7]. The definitions for mild (or minor) head injury are broader than that for mTBI; the history of head trauma is the primary basis for mild head injury, without necessarily demonstrating LOC, PTA or evidence of craniofacial trauma [1, 2, 4]. To the best of our knowledge, all of the studies examining the risk of DICH have been in mild head injury, while our patient more specifically met the criteria for mTBI.

Different potential mechanisms for post-traumatic DICH have been described. The risk of developing subdural haemorrhage due to shearing of the bridging veins, with associated cerebral atrophy of the elderly, may be exacerbated by a delayed compensatory hypertension for hypercapnia and local neurometabolic processes [9–11]. Estimating the traumatic forces involved in unwitnessed injuries may be difficult, especially if retrograde amnesia is also present. Currently used neurological and functional markers of severity in mTBI have limited predictive value, and imaging modalities in routine practice lack sensitivity for detecting very subtle changes for predicting the likelihood of DICH [12].

The small risk of DICH is a difficult dilemma from safety and resource allocation perspectives. In terms of safety, severe disability and death are real consequences of DICH, and very late presentations of DICH contribute to worse outcomes [1, 13, 14]. In terms of resource allocation, this is a rare complication of a common problem: the cost of routine hospitalizations for 24-hour observation and for repeat CT scans is difficult to justify with an estimated number needed to treat of up to 61 persons [15]. Further, even after 24 hours, it is possible to develop DICH [4]. The clinical relevance of diagnosing an asymptomatic DICH is questionable, especially if surgery is not needed [1, 4]. Recent reviews of this clinical problem have not recommended routine observations or another CT scan at 24 hours for DICH after the initial scan [16, 17], but observation for 24 hours was recommended in the recent Scandinavian guidelines, with a view to repeating the CT scan if clinically indicated [18]. The purpose of our case report is to continue these discussions regarding the management of the risk of DICH in persons on oral anticoagulation who sustain head injury, based on the lessons learned from a rehabilitation perspective.

## Case presentation

**Fig. 1:**
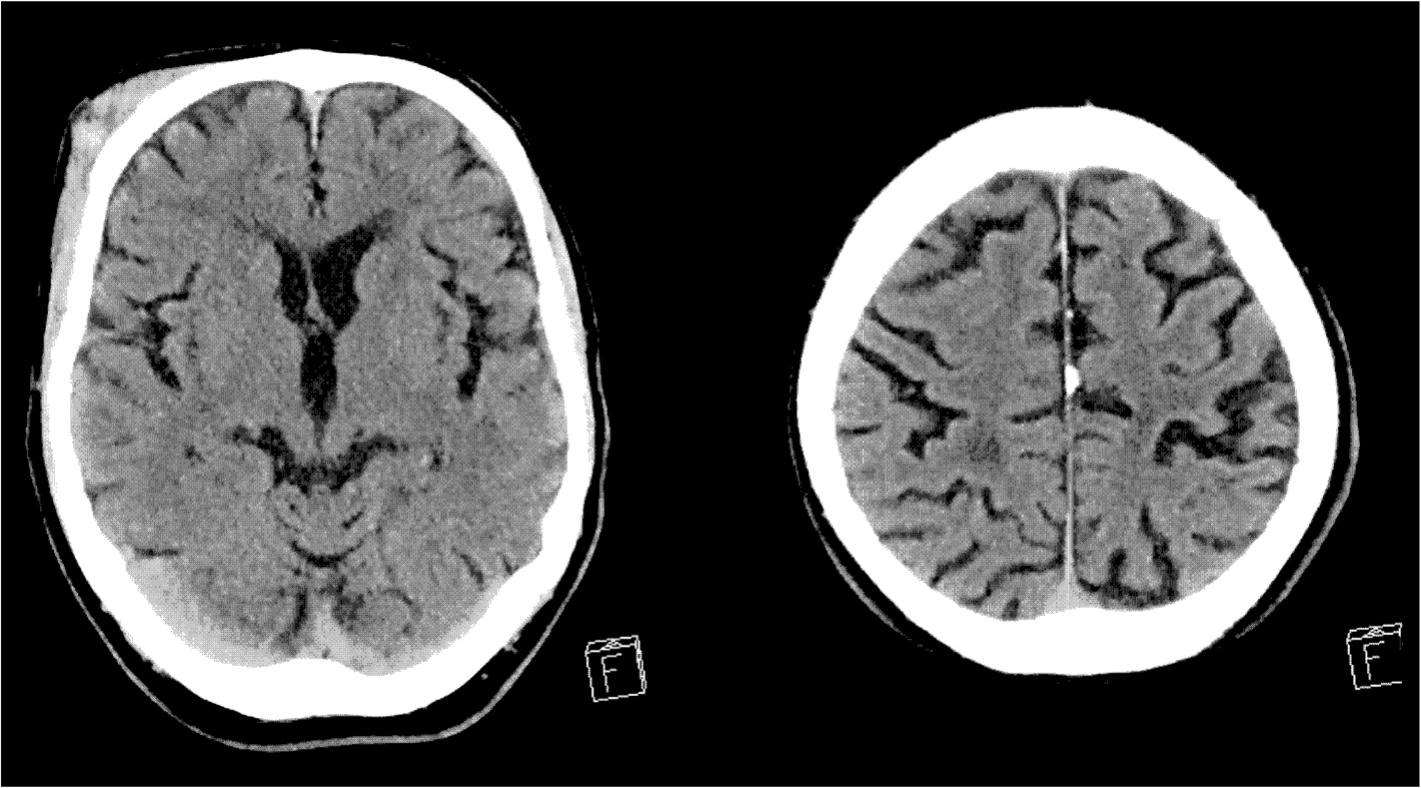
Computerised tomography scan at five hours from injury, with Glasgow Coma Scale score of 15, demonstrating right facial hematoma, but no acute intracranial hemorrhage

A 74-year-old Lebanese man living with his wife in Melbourne, Australia, who was previously independent with all activities of daily living, mobility and driving a car, sustained an unwitnessed fall resulting in mild TBI, with an initial GCS score of 15 on presentation. He was on warfarin and sotalol for chronic atrial fibrillation, with a target international normalized ratio (INR) of two to three, and a past history of transient ischaemic attack eight years earlier. He was also treated for hypertension. On the day of the injury, he was last seen in his garden pruning a tree on a ladder at a height of two meters. He had no recollection of immediate events leading up to the fall, or the fall itself. His wife noticed extensive right facial bruising when he entered the house, which led to the presentation to his usual local medical officer, who referred him on to the Emergency Department at the Royal Melbourne Hospital (Victoria, Australia), a major trauma centre. The initial CT scan of his brain was performed at five hours from the injury due to the above delays in presentation. This was non-diagnostic for intracranial hemorrhage (Fig. 1). His INR was elevated at 4.2. He was assessed by the neurosurgical team, and was admitted for a 24-hour observation period, with a planned repeat CT brain scan prior to discharge to home to the care of his family. His GCS score remained at 14 to 15 throughout the observation period, due to mildly confused speech at times throughout the night. He complained of a mild headache, which was managed with paracetamol as required.

**Fig. 2:**
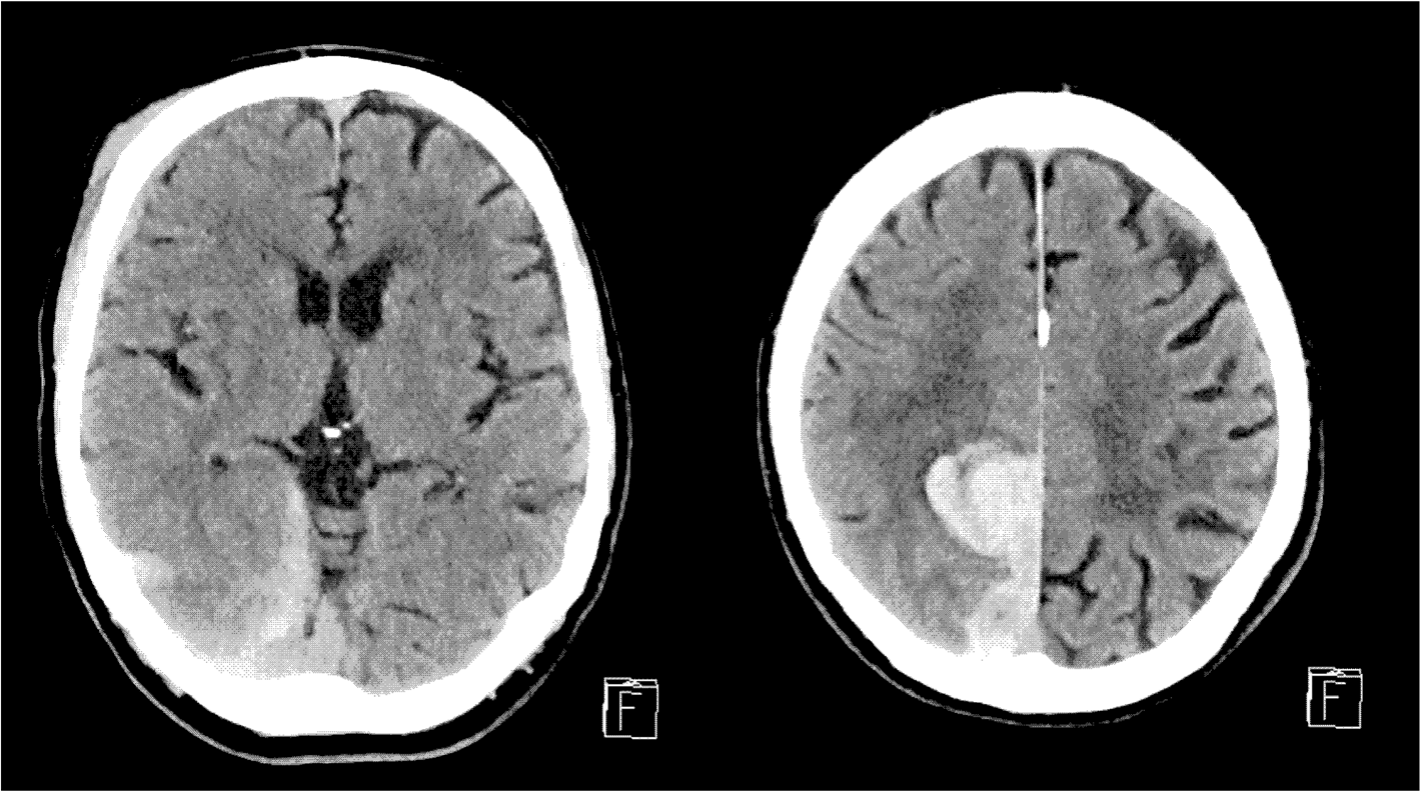
Computerised tomography scan at 24 hours from injury, with Glasgow Coma Scale score of 14 to 15, demonstrating right posteromedial parietal intraparenchymal hemorrhage (30×31×46mm) with vasogenic edema, subarachnoid and subdural extension, and right anterolateral subdural hematoma

On the following day, the repeat CT scan of his brain at 24 hours demonstrated a large right posteromedial parietal intraparenchymal hemorrhage with vasogenic edema, subarachnoid and subdural extension, and right anterolateral subdural hematoma (Fig. 2). He received urgent fresh frozen plasma, human prothrombin complex (Prothrombinex®-VF) (CSL Behring, Melbourne, Australia), and Vitamin K to reverse the anticoagulation, and he was admitted to the Stroke Care Unit, with a multidisciplinary team involved in his care. Further repeat scans did not demonstrate an extension of DICH, and surgical intervention was not required. During his admission, he sustained a fall due to left homonymous hemianopia, impulsivity and vertigo. He was found to have episodes of bradycardia, and his sotalol dosage was ceased. A subsequent CT scan of his brain did not demonstrate additional intracranial pathology. He was referred for inpatient rehabilitation.

On the 11th day after the injury, he was transferred to the Rehabilitation Unit at Royal Park Campus, Royal Melbourne Hospital, for multidisciplinary rehabilitation. On the 15th day, warfarin therapy was recommenced with close monitoring, with a target INR of two to three. His headache was minimal and his vertigo had resolved. He remained motivated and euthymic. A multidisciplinary assessment in the Rehabilitation Unit demonstrated the following problems: fatigue; mildly impaired arousal, attention, orientation, insight, processing speed, working memory and verbal learning; left homonymous hemianopia; decreased functioning, with minimal assistance of one person required for personal activities of daily living, including assisted showering and toileting; and decreased mobility with constant supervision required due to impulsivity and ongoing risk of falls, with minimal assistance of one person required for transfers and walking. He underwent an intensive three-week inpatient multidisciplinary rehabilitation program to address these problems.

By discharge, his fatigue and cognitive functioning had improved, and his family were educated on his supervision needs. A formal orthoptics assessment demonstrated full Bjerrum visual fields. However, in functional tasks and ambulation, he demonstrated left neglect and veered to the right side. He was independent with personal activities of daily living, and necessary home equipment was prescribed by his discharge. He was independent with transfers and ambulation indoors, without exercise tolerance limitations, but required supervision for outdoor mobility due to an ongoing visuospatial problem. He was also advised against driving on discharge for the same reason.

He attended a further two months’ outpatient multidisciplinary rehabilitation program at the Royal Park Campus, Royal Melbourne Hospital to address participation restrictions, with the goal of independent local community access. He was assessed in the outpatient clinic at three months from the injury, and by this stage, he was safely walking to the nearby shops alone, with increased independence in personal and domestic activities of daily living. However, the changed family roles as a result of the injury led to increasing caregiver stress, with ongoing driving restrictions and supervision needs. At his six-month review, he had no residual activity limitations, but his overall participation was restricted by his inability to return to driving.

## Discussion

We describe one case of DICH with limited applicability for the broader discussion in DICH. The management of the risk of DICH for persons on oral anticoagulation remains a difficult issue. The incidence of DICH in surveillance studies were variable, and has been reported to be 0.6% (n=4 out of 687) [1], 1% (n=4 out of 362) and 6% (n=5 out of 87) [4]. The first study used follow-up phone calls and medical records to detect cases, while the second and third studies (with higher incidences) used routine CT rescanning to detect cases at six hours and 24 hours from the injury, respectively. In all three studies, the case definition for head injury was non-specific, with no minimum criteria for the severity of the injury sustained. The timing of repeat scans also differed in surveillance studies, making it difficult to pool the data reliably. The question of whether very advanced age; an INR over three; LOC; or PTA increases risk of DICH remains unclear [1, 4], since persons with recognized poor prognostic factors for a poor outcome would presumably have had ICH detected on the initial scan. This is also complicated by the risk of DICH extending beyond 24 hours [4].

In light of the evidence, and as reflected by our case report, it is unsettling that current practices accept a small but real risk of DICH in mild head injury. A time-limited clear window of opportunity exists for clinicians to detect, or even to prevent DICH, but it is current routine practice for patients to remain anticoagulated, potentially at supratherapeutic INR levels. Initial management may differ if advanced imaging options are considered, such as magnetic resonance imaging (MRI), which may demonstrate subtle shear injuries not seen on CT scans [19]. However, the use of MRI for such a common presentation is just as difficult a problem from resource allocation and resource utilisation perspectives. Our understanding of acute injury variables contributing to DICH remains limited. Further, there is little known regarding the risk of DICH with novel oral anticoagulants, which are increasingly used [17]. Finally, for older persons who sustain mild head injuries from a fall, as in our patient, a thorough review of fall history and the individualized long term risk of further injuries should be carefully weighed against the overall indications for therapeutic anticoagulation.

Our patient was found to have significant neurological impairments from the DICH after his head injury; yet his cognitive and visuospatial deficits were subtle, and not detected as a significant deterioration, even by neurological observations. However, these problems resulted in activity limitations and participation restrictions compared with his pre-injury status, which significantly improved following the multidisciplinary rehabilitation. Is it relevant if a DICH is missed, is minimal and does not require surgical evacuation [16]? From the perspectives of providing patient-centred care to improve quality of life, it would seem relevant for patients and families to be given a clear diagnosis for their symptoms, as well as for any associated impairments to be identified, and for appropriate and timely rehabilitation care to be provided to prevent further complications and to treat any persistent deficits.

## Conclusions

Although small, the risk of DICH after mild head injury exists for persons on oral anticoagulation with warfarin. The optimal management for the risk of DICH remains unclear in the literature, and further studies are needed to clarify those who are at increased risk of DICH. Identification of DICH, even if not requiring surgical management, is highly relevant to providing patient-centered care, to functioning and to minimise disability.

## Consent

Written informed consent was obtained from the patient for publication of this case report and accompanying images. A copy of the written consent is available for review by the Editor-in-Chief of this journal.

## Abbreviations

CT: Computerised tomography
DICH: Delayed intracranial hemorrhage
GCS: Glasgow Coma Scale
INR: International normalized ratio
LOC: Loss of consciousness
MRI: Magnetic resonance imaging
mTBI: Mild traumatic brain injury
PTA: Post-traumatic amnesia
